# The Treatment of Eclampsia

**Published:** 1893-09

**Authors:** 


THE TREATMENT OF ECLAMPSIA.—Charpentier (*Annales de Gynecologie et d'Obstetrique*, vol. xxxix., 1893), in an elaborate report on the treatment of eclampsia, makes the following statements:

All women suffering from albuminuria are liable to eclampsia, and should be placed immediately on a milk diet, which is by far the best preventive. At all times during the convulsions the women become very much cyanosed, and from four to five hundred grammes of blood should be drawn, followed by the administration of chloral.

Allow labor to take place spontaneously, but at the same time hasten it as much as possible.

Sometimes the contractions of the uterus towards the close of labor become very feeble. In this case it is necessary to apply the forceps or perform version if the infant be alive, and to use the cephalotribe if it be dead.

Reserve the induction of labor for those cases where medical treatment has not completely relieved the aura.

Reject absolutely Cæsarean section and forced labor; deep incisions into the neck of the uterus and bleeding are preferable.

Gueniot claims that there are three forms of eclampsia:

1. Hypertoxic.
2. Neurasthenic.
3. The common form.

Against the toxæmia there is little to be done.

For the reflex excitability chloroform inhalation is the best treatment.

In certain very grave cases it is necessary to use chloral alone. Tarnier says the prognosis is always extremely grave and the mortality is about twenty-five per cent.—*Therapeutic Gazette.*

